# The role of vitamin C in melanoma cell death via activation of cytochrome C and TNF-α protein expression

**DOI:** 10.1038/s41598-025-18372-5

**Published:** 2025-10-06

**Authors:** Eva Krishna Sutedja, Tiara Rachmaputeri Arianto, Ghabrina Saraswati Elgianda, Endang Sutedja, Ronny Lesmana, Oki Suwarsa, Reti Hindritiani, Pati Aji Achdiat, Hendra Gunawan, Budi Setiabudiawan

**Affiliations:** 1 Department of Dermatology and Venereology, Faculty of Medicine, Universitas Padjajaran – Dr. Hasan Sadikin Hospital, Bandung, West Java Indonesia; 2 Department of Physiology, Faculty of Medicine, Universitas Padjajaran – Dr. Hasan Sadikin Hospital, Bandung, West Java Indonesia; 3 Department of Child Health, Faculty of Medicine, Universitas Padjajaran – Dr. Hasan Sadikin Hospital, Bandung, West Java Indonesia

**Keywords:** Apoptosis, Cytochrome c, Extrinsic pathway, Intrinsic pathway, TNF-α, Vitamin c, Cancer, Cell biology, Drug discovery, Oncology

## Abstract

This study investigates the effect of vitamin C on cytochrome c and TNF-α expression, representing intrinsic and extrinsic apoptotic pathways in B16-F10 melanoma cells. B16-F10 melanoma cells (ATCC^®^ CRL-6475™) were treated with vitamin C for 12 and 24 h. Expression of cytochrome c, TNF-α, caspase-3, and caspase-9 proteins was evaluated using in-cell western (ICW) assay. RT-qPCR was used to assess gene expression of Caspase-8, IL-6, PARP-γ, and TGF-β1. Morphological signs of cell death appeared within 12 h. A significant increase in cytochrome c expression at 3,000 µM concentrations of vitamin C was observed (*p* = 0.002), along with increases at 3,500 µM (*p* = 0.023), 4,000 µM (*p* = 0.0001), and 5,000 µM (*p* = 0.0001). Caspase-3 expression significantly increased at 3,000 µM (*p* = 0.012) and 5,000 µM (*p* = 0.048), while caspase-9 was not significant. TNF-α expression also increased significantly from 3,000 to 5,000 µM (all *p* < 0.01). Caspase-8 and PARP-γ mRNA levels were upregulated at 3,000 and 5,000 µM (*p* < 0.05). IL-6 and TGF-β1 changes further supported apoptosis activation. Vitamin C induces apoptosis in B16-F10 melanoma cells via intrinsic and extrinsic pathways, particularly at 3,000 and 5,000 µM concentrations, as shown by cytochrome c, TNF-α, and caspase-3 expression.

**Supplementary Information:**

The online version contains supplementary material available at 10.1038/s41598-025-18372-5.

## Introduction

Melanoma is a type of cancer emerging from melanocytes, a pigment-producing cell predominantly found in the skin, uvea, inner ear, and meninges^1,2^. Melanoma is regarded as an aggressive tumor with significant metastatic potential. Despite a mortality rate of only about 20%, melanoma is responsible for 80% of skin cancer-related deaths worldwide, largely because of its strong metastatic potential^2–4^. According to recent statistics, there are approximately 300,000 new cases and over 60,000 deaths annually^5^. The highest incidence was reported in Australia and New Zealand with 35 new cases per 100,000 population per year^6^. Melanoma is more prevalent in females compared to males and in people aged 40–59 years old^7^. The prevalence typically remains steady during the age range, and disease progressiveness tend to increase in the ≥ 60 years old population^8^. The etiology of melanoma is multifactorial, involving environmental (ultraviolet radiation/ UVR) and genetic factors (Fitzpatrick skin phototype, number of melanocytic nevi, previous or family history of melanoma or other skin cancers, being immunosuppressed, or having genetic mutation)^9,10^. Histopathologically, there are 4 subtypes of melanoma: superficial spreading melanoma (SSM), nodular melanoma (NM), lentigo malignant melanoma (LMM), and acral lentiginous melanoma (ALM)^11^. Melanoma can be treated using single or combination therapy, depending on its severity. Treatment modalities include surgical excision, chemotherapy, photodynamic therapy, immunotherapies, biochemotherapy, and targeted therapy^6,12^.

Vitamin C, also known as ascorbic acid, is a water-soluble micronutrient with antioxidant properties, collagen biosynthesis, energy metabolism, and iron absorption. Previous studies have demonstrated the anti-cancer properties of vitamin C in cases of breast, lung, renal, colon, and pancreatic cancers, as well as leukemia^13,14^. Not only capable of protecting normal tissues from free radical damage, vitamin C is also capable of protecting cancer cells from chemotherapy/radiotherapy-induced reactive oxygen species (ROS). It is cytoprotective at low concentrations but cytotoxic at high concentrations^14^. Its capacity to produce hydrogen peroxide (H_2_O_2_) in the extracellular fluid surrounding the tumor is correlated with its lethal action. Vitamin C acts as a pro-oxidant in the presence of catalytic metals, causing oxidative stress and ROS production, which then leads to apoptosis^15^. Apoptosis is divided into three pathways: the intrinsic, extrinsic, and perforin/granzyme pathways. Cytochrome c is a key regulator of the intrinsic apoptosis pathway. Meanwhile, TNF-α induces apoptosis via the extrinsic pathway by activating death receptors in the cell surface. Caspase-3 serves as a key mediators of apoptosis, cleaving cellular proteins in response to specific death stimuli^16^.

Cell culture is a laboratory technique for studying human physiology and metabolism^17^. The B16-F10 melanoma cell line, originally derived from C57BL/6J mice^18^, is widely used in cancer research due to its high metastatic potential. This model is particularly suitable for evaluating adjuvant therapies in melanoma^19,20^. Based on these prior understandings, the objective of this study is to investigate the effect of vitamin C on cytochrome c protein in the intrinsic apoptosis pathway and TNF-α ligand expression in the extrinsic apoptosis pathway of melanoma B16-F10 cell line. Other apoptosis-specific proteins, such as caspase-3, were used to confirm that cytochrome c and TNF-α expression is part of the apoptosis process.

## Materials and methods

### Culture of melanoma cell line

The melanoma B16-F10 cell line (ATCC^®^ CRL-6475TM) was cultivated in RPMI medium with 10% fetal bovine serum (FBS) and 1% penicillin-streptomycin (Gibco^®^) in a 10 cm Petri dish at 37 °C with 5% CO₂. Cells were monitored until reaching 80–90% confluence. Approximately 10⁵ cells were cultured in 96-well plates and divided into 19 groups: RPMI medium, 2% dimethyl sulfoxide (DMSO) as vitamin C solvent due to stability reasons and consistency with other treatments, cisplatin 40 µM as a positive control, and vitamin C at doses ranging from 500 µM to 40,000 µM. Effects were observed at 12 h and 24 h. All test compounds were prepared in DMSO, with the final DMSO concentration in all treatment groups adjusted to 2%. As a separate vehicle control containing 2% DMSO alone was not included, the possibility of DMSO-induced cytotoxicity cannot be entirely excluded.

### Microtetrazolium test

Cell viability was assessed using the MTT assay. Cells and tested compounds were prepared in 96-well plates (100 µL/well). Presto Blue and 10 µL of MTT solution (0.45 mg/mL) were added. After 1–4 h of incubation at 37 °C, 100 µL solvent (SDS in hydrochloric acid, isopropanol, and 2% DMSO) was added to dissolve formazan crystals. Absorbance at 575 nm was measured via spectrophotometry. Higher absorbance indicated greater cell viability. The hue of a dying cell is blue whereas that of a living cell is purple. The outcome was determined by comparing the color of the cell line treated with only the medium to the cell line supplemented with vitamin C. Cell viability was calculated as a percentage relative to the untreated control group, which was designated as 100% viability. Higher absorbance values reflect greater metabolic activity, indicating higher relative viability.

### In-cell western assay

Levels of cytochrome c, TNF-α, caspase-9, and caspase-3 were measured using ICW assay. Cells were cultured in 96-well plates for 24 h. Fixation was done using 3.7% formaldehyde for 20 min, followed by permeabilization using 0.1% Triton X-100. Cells were blocked with TBS for 30 min. After disposal, 50 µL of primary antibodies (Cytochrome C, TNF-α, caspase-9, and caspase-3) were added and incubated for 2 h.

After incubation with primary antibodies, well plates were rinsed with wash buffer and incubated with fluorochrome-conjugated secondary antibodies for 1 h in the dark. After final rinsing, plates were dried and scanned using an LiCor^®^ machine.

### Statistical analysis

Statistical analyses were performed using SPSS 25.0 and GraphPad Prism 8. Experiments were repeated twice (*n* = 2) to minimize bias. Data were tested for normality (Shapiro-Wilk test) and homogeneity (Levene’s test). One-way ANOVA was used for normally distributed data, while the Kruskal–Wallis test was used otherwise. Post-hoc tests included LSD for ANOVA and the Mann-Whitney U test for Kruskal-Wallis. Statistical significance was set at *p* ≤ 0.05.

## Results

The treatment resulted in more than 50% cell death at each dose after 12 h of observation, as indicated by a reduced number of cell confluence and noticeable shape changes. To prevent excessive cell death, further observations beyond 12 h were not conducted.

**Fig. 1:**
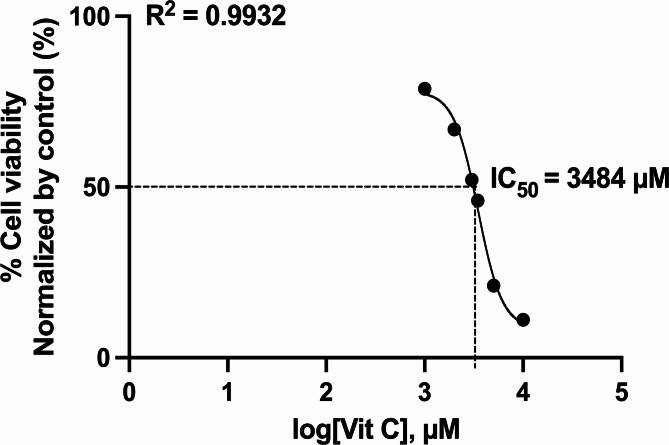
IC_50_ analysis using MTT method.

The MTT assay was used to determine the half-maximal inhibitory concentration (IC_50_), which was used to calculate the dosage of vitamin C in the ICW assay. The post-treated melanoma cell line was treated with presto blue reagent. More than 50% cell death was observed at a dose ≥ 3.484 µM. The IC_50_ values were determined using nonlinear regression (GraphPad Prism) effects in the lower micromolar range are shown in Fig. 1.

**Fig. 2:**
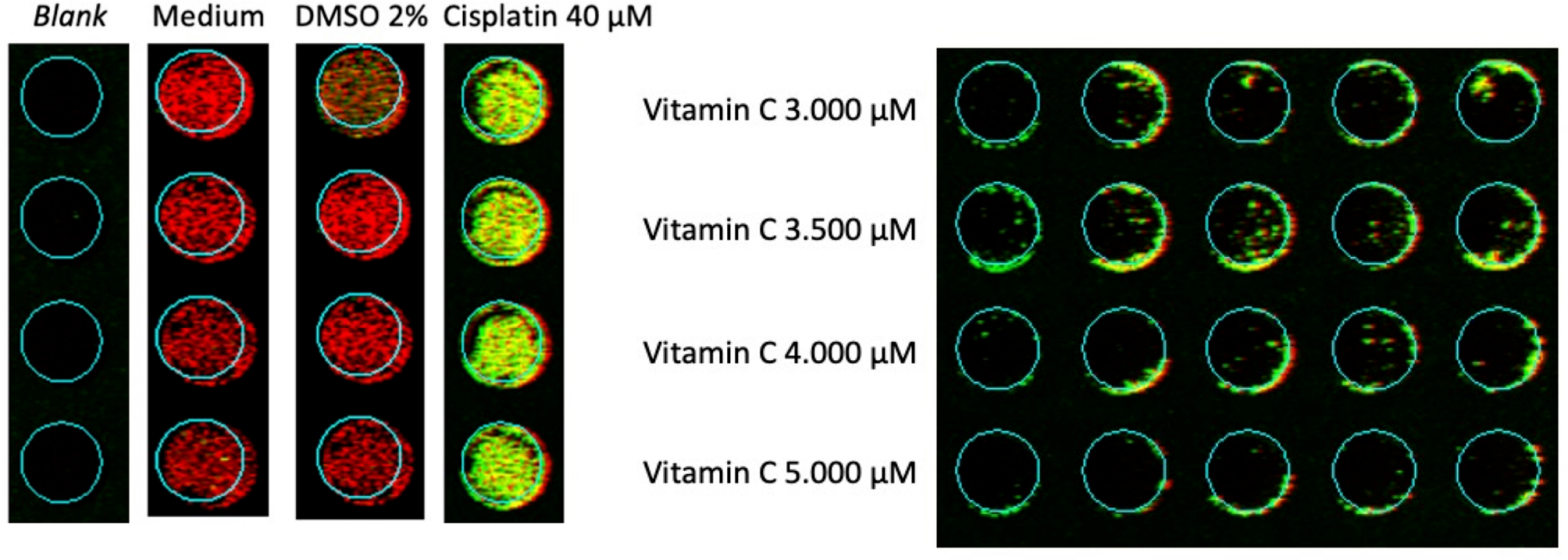
Protein expressions 12 h after treatment: Cytochrome c (green fluorescence), Cell Tag™ 700 (red fluorescence), and combination (yellow fluorescence).

**Fig. 3:**
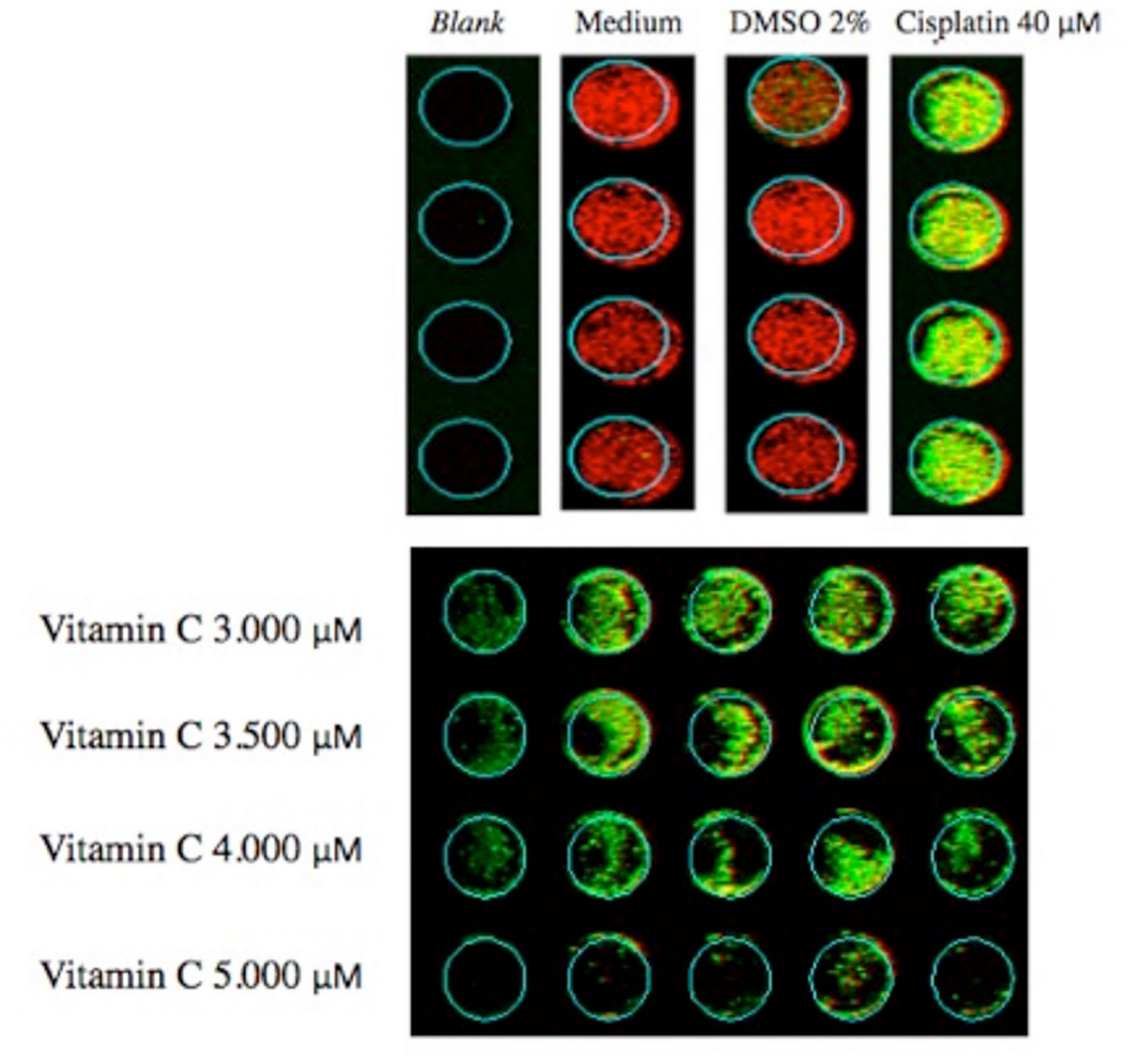
Protein expressions 12 h after treatment: TNF-α (green fluorescence), Cell Tag™ 700 (red fluorescence), and combination (yellow fluorescence).

An in-cell western assay was used to examine cytochrome c and TNF-α protein expression. Tag™ 700 which served as an internal control, was also added and showed a red fluorescence. Meanwhile, the cytochrome c and TNF-α protein was green. If both green and red were expressed together, the fluorescence would appear yellow. Cytochrome C protein expression in this experiment is shown in Fig. 2 and TNF-α protein expression is depicted in Fig. 3. The figure shows the red color in 2% DMSO as a control towards vitamin C, indicating no increase in cytochrome c and TNF-α expression. The cisplatin cell lineage is green, indicating an increase in cytochrome c expression. At doses ranging from 3.000 µM to 5.000 µM, vitamin C-treated cells were yellowish-green in color.

**Fig. 4:**
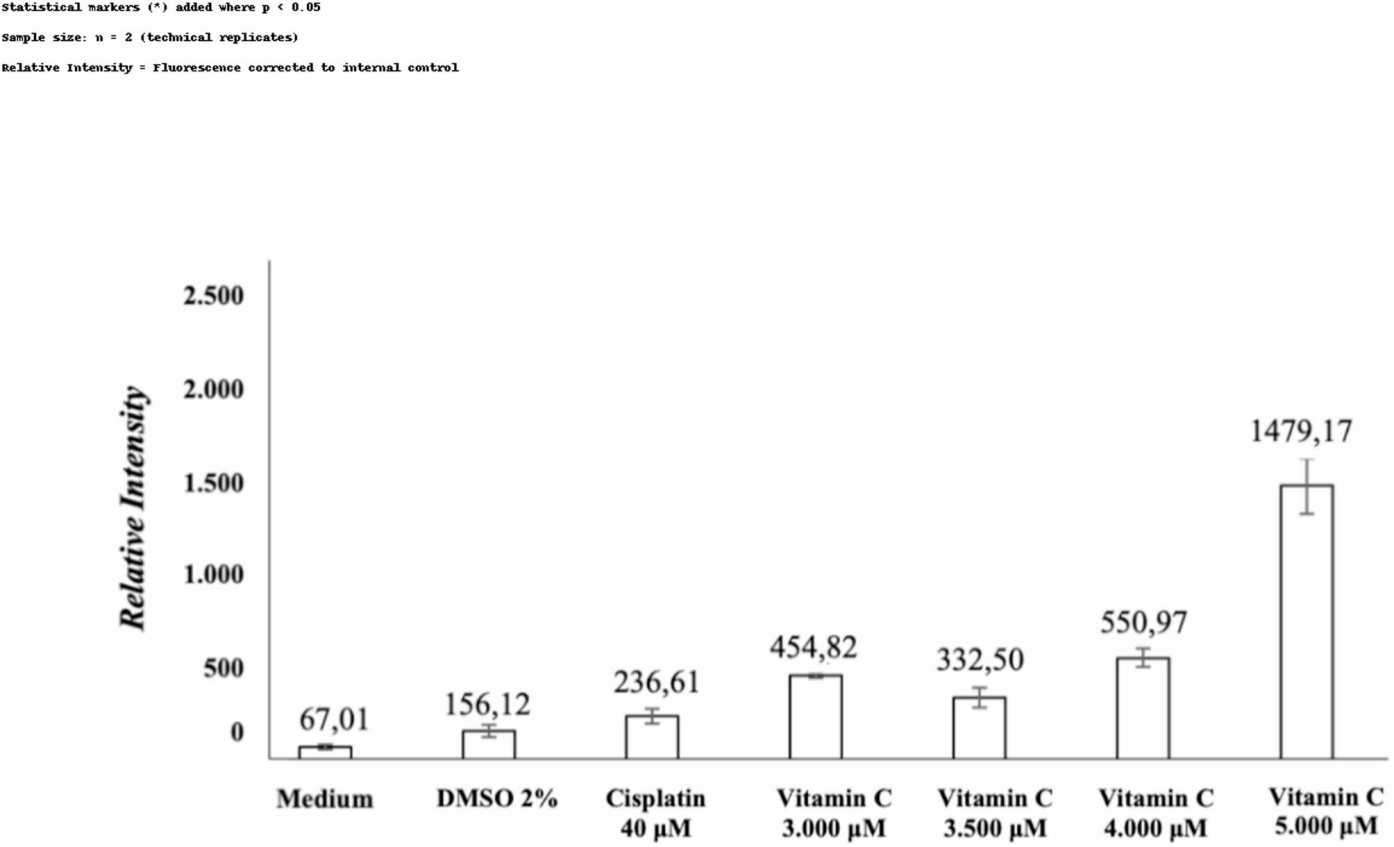
Mean comparison of cytochrome c expressions on melanoma B16-F10 cell line 12 h post-treatment.

This study also compared cytochrome c protein expression under different conditions with vitamin C: medium-only, 2% DMSO, and cisplatin, as shown in Tables 1, 2, and 3 respectively. All comparisons had a normal distribution and homogenous variants; hence, one-way ANOVA and post-hoc LSD were used. When comparing medium-only and vitamin C, significant differences were found at doses of 3,000 µM (*p* = 0.002), 3,500 µM (*p* = 0.023), 4,000 µM (*p* = 0.0001), and 5,000 µM (*p* = 0.0001). Similarly, in the comparison between vitamin C and 2% DMSO, significant differences emerged at doses of 4,000 µM (*p* = 0.012) and 5,000 µM (*p* = 0.0001). In comparison to cisplatin, significant differences were observed at 4,000 µM (*p* = 0.009) and 5,000 µM (*p* = 0.0001). These comparisons are shown in Fig. 4.

**Table 1:** The comparison of cytochrome c expressions on melanoma B16-F10 cell line using medium and vitamin C.

Variable	*N*	Cytochrome c protein expression			*p*-value	
		Mean ± Std	Median	Range (min-max)		
Medium	3	67.01 ± 25.278	62.90	44.05–94.10	**0**.002*	
Vitamin C 3.000 µM	4	454.82 ± 19.959	449.05	438.13–483.03		
Medium	3	67.01 ± 25.278	62.90	44.05-94.10	**0**.023*	
Vitamin C 3.500 µM	4	332.50 ± 106.634	363.28	188.56-414.89		
Medium	3	67.01 ± 25.278	62.90	44.05–94.10	**0**.0001**	
Vitamin C 4.000 µM	4	550.97 ± 104.053	542.48	433.53–685.40		
Medium	3	67.01 ± 25.278	62.90	44.05–94.10	**0**.0001**	
Vitamin C 5.000 µM	4	1479.17 ± 297.419	1351.69	1290.18–1923.14		

Statistically significant at *p* value ≤ 0.05 (*) and ≤ 0.01 (**)

**Table 2:** The comparison of cytochrome c expressions on melanoma B16-F10 cell line using 2% DMSO and vitamin C.

Variable	*N*	Cytochrome c protein expression			*P*-value
		Mean ± Std	Median	Range (min-max)	
Cisplatin 40 µM	3	236.61 ± 67.214	267.87	159.46–282.51	**0**.**055**
Vitamin C 3.000 µM	4	454.82 ± 19.959	449.05	438.13–483.03	
Cisplatin 40 µM	3	236.61 ± 672.14	267.87	159.46–282.51	**0**.**380**
Vitamin C 3.500 µM	4	33250 ± 106634	363.28	188.56-414.89	
Cisplatin 40 µM	3	236.61 ± 67.214	267.87	159.46–282.51	**0**.009*
Vitamin C 4.000 µM	4	550.97 ± 104.053	542.48	433.53–685.40	
Cisplatin 40 µM	3	236.61 ± 67.214	267.87	159.46–282.51	**0**.0001**
Vitamin C 5.000 µM	4	1479.17 ± 297.419	1351.69	1290.18-1923.14	

Statistically significant at *p* value *≤* 0.05 (*) and *≤* 0.01 (**).

**Table 3:** The comparison of cytochrome c expressions on melanoma B16-F10 cell line using cisplatin and vitamin C.

Variable	*N*	Cytochrome c protein expression			*p*-value
		Mean ± Std	Median	Range (min-max)	
2% DMSO	3	156.12 ± 56.746	171.43	93.29–203.64	**0**.012*
Vitamin C 3.000 µM	4	454.82 ± 19.959	449.05	438.13–483.03	
2% DMSO	3	156.12 ± 56.746	171.43	93.29–203.64	**0**.**115**
Vitamin C 3.500 µM	4	332.50 ± 106.634	363.28	188.56–414.89	
2% DMSO	3	156.12 ± 56.746	171.43	93.29–203.64	**0**.002*
Vitamin C 4.000 µM	4	550.97 ± 104.053	542.48	433.53–685.40	
2% DMSO	3	156.12 ± 56.746	171.43	93.29–203.64	**0**.0001**
Vitamin C 5.000 µM	4	1479.17 ± 297.419	1351.69	1290.18-1923.14	

Statistically significant at *p* value *≤* 0.05 (*) and *≤* 0.01 (**).

**Fig. 5:**
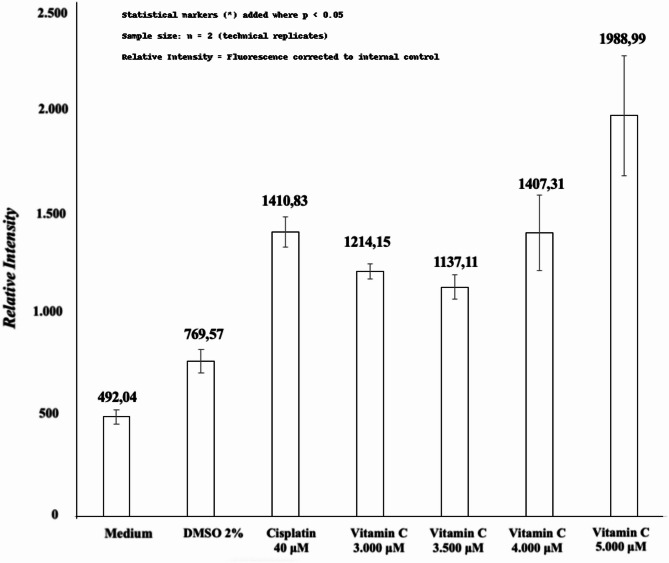
Mean comparison of TNF-α expressions on melanoma B16-F10 cell line 12 h post-treatment.

Furthermore, this study also contrasted TNF-α protein expression under various conditions with vitamin C: medium-only, 2% DMSO, and cisplatin, as presented in Tables 4, 5, and 6. All comparisons demonstrated normal distribution and homogeneous variance, enabling the utilization of one-way ANOVA and post-hoc LSD tests. When comparing medium-only and vitamin C, significant differences were found at doses of 3,000 µM (*p* = 0.005), 3,500 µM (*p* = 0.011), 4,000 µM (*p* = 0.001), and 5,000 µM (*p* = 0.0001). Similarly, in the comparison between vitamin C and 2% DMSO, significant differences emerged at doses of 4,000 µM (*p* = 0.012) and 5,000 µM (*p* = 0.0001). In comparison to cisplatin, significant differences were observed at 5,000 µM (*p* = 0.021), but not at lower doses. These comparative findings are depicted in Fig. 5.

**Table 4:** The comparison of cytochrome c expressions on melanoma B16-F10 cell line using cisplatin and vitamin C.

Variable	*N*	TNF-α protein expression			*P*-value	
		Mean ± Std	Median	Range (min-max)		
Medium	3	492.05 ± 60.520	473.02	443.33–559.80	**0**.005**	
Vitamin C 3.000 µM	4	1214.16 ± 73.889	1228.43	1112.93-1286.84		
Medium	3	492.05 ± 60.520	473.02	443.33–559.80	**0**.011*	
Vitamin C 3.500 µM	4	1137.12 ± 123.418	1155.68	972.31-1264.80		
Medium	3	492.05 ± 60.520	473.02	44.05–94.10	**0**.001*	
Vitamin C 4.000 µM	4	1407.32 ± 376.127	1395.39	1046.33-1823.87		
Medium	3	492.05 ± 60.520	473.02	44.05–94.10	**0**.0001**	
Vitamin C 5.000 µM	4	1988.99 ± 594.579	2023.38	1253.18-2656.02		

Statistically significant at *p* value *≤* 0.05 (*) and *≤* 0.01 (**).

**Table 5:** The comparison of TNF-α expressions on melanoma B16-F10 cell line using 2% DMSO and vitamin C.

Variabel	*N*	TNF-α protein expression			*P*-value
		Mean ± Std	Median	Range (min-max)	
Cisplatin 40 µM	3	1410.84 ± 132.314	1395.39	1286.93-1550.20	**0**.**401**
Vitamin C 3.000 µM	4	1214.16 ± 73.889	1228.43	1112.93-1286.84	
Cisplatin 40 µM	3	1410.84 ± 132.314	1395.39	1286.93-1550.20	**0**.**246**
Vitamin C 3.500 µM	4	1137.12 ± 123.418	1155.68	972.31-1264.80	
Cisplatin 40 µM	3	1410.84 ± 132.314	1395.39	1286.93-1550.20	**0**.**988**
Vitamin C 4.000 µM	4	1407.32 ± 376.127	1379.54	1046.33-1823.87	
Cisplatin 40 µM	3	1410.84 ± 132.314	1395.39	1286.93-1550.20	**0**.021*
Vitamin C 5.000 µM	4	1988.99 ± 594.579	2023.38	1253.18-2656.02	

Statistically significant at *p* value *≤* 0.05 (*) and *≤* 0.01 (**).

**Table 6:** The comparison of TNF-α expressions on melanoma B16-F10 cell line using cisplatin and vitamin C.

Variable	*N*	TNF-α protein expression			*P*-value	
		Mean ± Std	Median	Range (min-max)		
2% DMSO	3	769.57 ± 101.865	795.80	657.16–855.76	**0**.**067**	
Vitamin C 3.000 µM	4	1214.16 ± 73.889	1228.43	1112.93-1286.84		
2% DMSO	3	769.57 ± 101.865	795.80	657.16–855.76	**0**.**125**	
Vitamin C 3.500 µM	4	1137.12 ± 123.418	1155.68	972.31-1264.80		
2% DMSO	3	769.57 ± 101.865	795.80	657.16–855.76	**0**.012*	
Vitamin C 4.000 µM	4	1407.32 ± 376.127	1379.54	1046.33-1823.87		
2% DMSO	3	769.57 ± 101.865	795.80	657.16–855.76	**0**.0001**	
Vitamin C 5.000 µM	4	1988.99 ± 594.579	2023.38	1253.18-2656.02		

Statistically significant at *p* value *≤* 0.05 (*) and *≤* 0.01 (**).

In the expression of caspase-3, p *≤* 0.05 were found in the comparison between medium-only and supplemental vitamin C at doses 3,000 µM (*p* = 0.012) and 5,000 µM (*p* = 0.048). In contrast, the expression of caspase-9 is not statistically significant in all of vitamin C doses.

When comparing cytochrome c, caspase-3, and caspase-9 expression following vitamin C administration, one-way ANOVA and post-hoc LSD were used at doses of 3,000 µM, 3,500 µM, and 4,000 µM. A *p* value of *≤* 0.05 were observed for caspase-3 at vitamin C doses of 3,000 µM (*p* = 0.0001), 3,500 µM (*p* = 0.023), and 4,000 µM (*p* = 0.029), but not in comparison to caspase-9. Kruskal Wallis with post-hoc Mann Whitney test was used for a vitamin C dose of 5,000 µM because of its non-normal distribution. *p* value of *≤* 0.05 were found in caspase-3 at a dose of 5,000 µM (*p* = 0.047), but not in comparison to caspase-9.

In addition to protein analysis, gene expression was analyzed using RT-qPCR. The results revealed a significant upregulation of Caspase-8 and PARP-γ gene expression in cells treated with 3,000 µM and 5,000 µM of vitamin C (*p* < 0.05), further supporting the involvement of the extrinsic apoptosis pathway. TGF-β1 expression increased significantly, particularly at 3,500 µM and 4,000 µM (*p* = 0.0045), while IL-6 expression was notably modulated, indicating a role in inflammatory and apoptotic signaling. These findings were consistent with the protein-level changes observed through ICW assays, particularly the expression of caspase-3 and TNF-α.

## Discussion

This study demonstrated that vitamin C induces apoptosis in the melanoma B16-F10 cell line. The outcome is comparable to the findings of Chen et al., which showed that the increasing B-cell lymphoma protein-2 (Bcl-2) associated x (Bax)/Bcl-2 ratio on melanoma A375 might experience apoptosis as induced by 600 µM and 1,400 µM vitamin C through the mitochondria pathway^21^ Yang et al. also showed that 1,000 µM and 5,000 µM vitamin C reduce the viability of melanoma A2058 cell^14^.

In the current study, IC_50_ was found to occur at a dose of *≥* 3,484 µM. The IC_50_ is effective for measuring the dosage required to inhibit physiological function, as indicated by > 50% cell death^22,23^. This examination assessed cell metabolic activity as an indicator of cell viability, proliferation, and cytotoxicity^22,24^. Based on the mean IC_50_, the following vitamin C doses were chosen for the ICW assay: 3,000 µM, 3,500 µM, 4,000 µM, and 5,000 µM. The cell line that was administered medium only and 2% DMSO showed red fluorescence, indicating a live melanoma B16-F10 cell line. Meanwhile, cells treated with 40 µM cisplatin became green as a sign of cell death. After quantification, the mean of the medium-only cell line was 67.01 while of the 2% DMSO was 156.12. The mean of the cells treated with cisplatin was 236.61, while the mean vitamin C level was higher. The means at vitamin C doses of 3,000 µM, 3,500 µM, 4,000 µM, and 5,000 µM were 454.82, 332.50, 550.97, and 1479.17 respectively. Post-hoc analysis revealed p *≤* 0.05 in terms of cytochrome c expression when comparing medium-only with all doses of vitamin C. Similarly, when 2% DMSO was compared with vitamin C, all doses except 3,500 µM were statistically significant. This demonstrated that vitamin C administration in B16-F10 melanoma cell lines activated the intrinsic apoptotic pathway. This result was supported by previous studies, which showed that apoptosis was induced after vitamin C administration in breast and colon cancers, marked by an increase in cytochrome c^25,26^. To prove that cytochrome c expression was part of the apoptosis process, apoptosis-specific proteins, such as caspase-9 and caspase-3, were used for confirmatory comparison. Subsequent analysis indicated a significance level of *p* < 0.05 for TNF-α expression when comparing the medium-only group to all vitamin C doses. Likewise, when comparing the 2% DMSO group with vitamin C doses, all doses except 5,000 µM showed statistical significance. This underscores that the introduction of vitamin C to B16-F10 melanoma cell lines also triggered the extrinsic apoptotic pathway. Prior research findings aligned with this outcome, demonstrating that vitamin C administration induced apoptosis in breast cancer cases, as evidenced by elevated TNF-α levels^27^. To establish the role of TNF-α expression in the apoptotic cascade, the study employed apoptosis-specific proteins like caspase-3 for corroborative analysis.

Each treatment resulted in different levels of protein expression. The mean expression levels of cytochrome c, TNF-α, and caspase-3 were lower in the medium-only treatment than in other treatments. Based on LSD analysis test, significant results were found on protein cytochrome c and caspase-3 expressions toward the cell line given medium-only with all and most of the vitamin C dose, respectively. To the best of our knowledge, no study has demonstrated whether RPMI medium alone influences apoptotic processes. In the 2% DMSO group, the mean expression of cytochrome c, TNF-α, and caspase-3 was elevated compared to the medium-only group, while caspase-9 was decreased. However, none of these proteins had a *p* value ≤ 0.05 which indicated statistical insignificance. In contrast to this study, Dludla et al., showed that > 1% DMSO may induce MOP, which causes apoptosis^27^. Similarly, Galvao et al., proved that > 10% DMSO may cause apoptosis through ROS^28^. The current use of 2% DMSO may have influenced the increase in proapoptotic protein expression. Compared to vitamin C, administration of cisplatin to B16-F10 melanoma cells, used as a positive control for cell death, in comparison to 4.000 µM and 5.000 µM vitamin C, was found to significantly increase cytochrome c expression but did not generate statistically significant outcomes regarding TNF-α or caspase-3 protein expression when juxtaposed with the medium-only control. A similar finding was found for caspase-9 expression in comparison with 3.000 µM vitamin C. However, there was no significant difference in the expression of these proteins when compared to the medium only. A previous study conducted on an ovarian cancer cell line found that cisplatin could induce the pro-apoptotic protein Bax and B-cell lymphoma antagonist/killer (BAK) which activates caspase-9 and caspase-3. This study also showed that cisplatin might induce ROS, which would cause the release of cytochrome c from the mitochondria and induce pro-apoptotic TNF-α and PKC-δ protein expression^29–31^. Contrary to this result, Gonzalez et al., demonstrated that cisplatin did not always cause apoptosis. The ability of high-dose cisplatin to reduce ATP levels, leading to necrosis, may be the reason for this. It was also mentioned that although one proapoptotic protein was activated, apoptosis did not always occur, and cisplatin may induce another type of cell death^31^. This was supported by Lieberthal et al., who demonstrated the dose-dependent properties of cisplatin. Cisplatin promoted necrosis at high dose (800 µM), whereas it induced apoptosis at low dose (8 µM)^32^. The non-statistically specific result on protein expression of cytochrome c, caspase-9, and caspase-3 might be due to the shifting of cell death, from apoptosis to necrosis. At high doses (312 µM), cisplatin can diminish intracellular ATP concentration, resulting in necrosis. Moreover, even when one pro-apoptotic protein is activated, apoptosis is not guaranteed, leading to alternative forms of cell death^33^. Thus, cisplatin could only be used as cell death-positive control but not as apoptosis-positive control.

The current experiment did not evaluate time-dependent protein expression, because only one plate of ICW was used for each tested protein. This may have affected the insignificant results. Another factor that may contribute to this effect is the inhibition of apoptosis in caspase-9. Eiga et al. identified that cancer cells have several protective systems to avoid apoptosis, such as the survivin protein. The target of this protein was caspase-9^33^. Another study on caspase-9 expression in cell lines showed several factors which could inhibit caspase-9, including casein kinase (CK) 2, among others^34^. This apoptosis inhibitor was found in murine cells, but not humans^35^. In this study, B16-F10 was used, a murine melanoma culture cell from mice C57BL/6J. Comparison of caspase-3 expression between medium-only and vitamin C 3,000 µM (*p* = 0.012) and 5,000 µM vitamin C (*p* = 0.048) showed statistically significant results. This indicated that apoptosis occurred, and proved the relationship of vitamin C administration and an increase in cytochrome c and TNF-α expression. A comparison of caspase-3 and cytochrome expression in vitamin C showed significant results at doses of 3,000 µM (*p* = 0.0001), 3,500 µM (*p* = 0.029), 4,000 µM (*p* = 0.029), and 5,000 µM (*p* = 0.047). A study by Chen et al. used caspase-3 as an apoptosis indicator, where caspase-3 was found to be increased in melanoma A375 cells^36^. The different results between caspase-9 and caspase-3 might be due to the time required from the beginning of apoptosis before caspase-3 activation. The time needed for caspase-9 to expressed was relatively short, less than 5 min^37^. Caspase-3 could be expressed in cells for 8 h^37^. Additionally, caspase-3 could also be expressed through perforin/granzyme pathways^38^. Moreover, gene expression analysis using RT-qPCR revealed elevated levels of caspase-8, IL-6, PARP-γ, and TGF-β1 following vitamin C treatment. This corroborates the protein-level findings and supports the involvement of both intrinsic and extrinsic apoptotic pathways. Caspase-8, as a marker of the extrinsic pathway, along with upregulated PARP-γ and TGF-β1 expression, further strengthens the hypothesis of vitamin C-induced apoptosis in melanoma cells^39,40^.

This study had some limitations. The duration of the experiment was constrained, rendering comparisons of time-dependent protein expressions impossible. Therefore, further time-dependent studies are required to achieve the maximum results. Moreover, future in-vivo studies should be conducted to examine the effect of vitamin C on melanoma.

## Conclusion

Taken together, this study concluded that vitamin C administration to the melanoma B16-F10 cell line at doses of 3,000 and 5,000 µM had an impact on the increase in cytochrome c and TNF-α expression along with caspase-3 as a proof of its activity in the intrinsic and extrinsic apoptosis pathway. These results suggest a potential adjuvant treatment strategy of Vitamin C for melanoma.

## Supplementary Information

Below is the link to the electronic supplementary material.

Supplementary Material 1

## Data Availability

No datasets were generated or analysed during the current study.
