# Supplementary material for: The role of vitamin C in melanoma cell death via activation of cytochrome C and TNF-α protein expression
Source: Sci Rep. 2025 Oct 6;15:34733. doi: 10.1038/s41598-025-18372-5 (PMC12500876; doi:10.1038/s41598-025-18372-5)
Supplement: Supplementary file 1 — Supplementary Material 1 [file 41598_2025_18372_MOESM1_ESM.pdf]

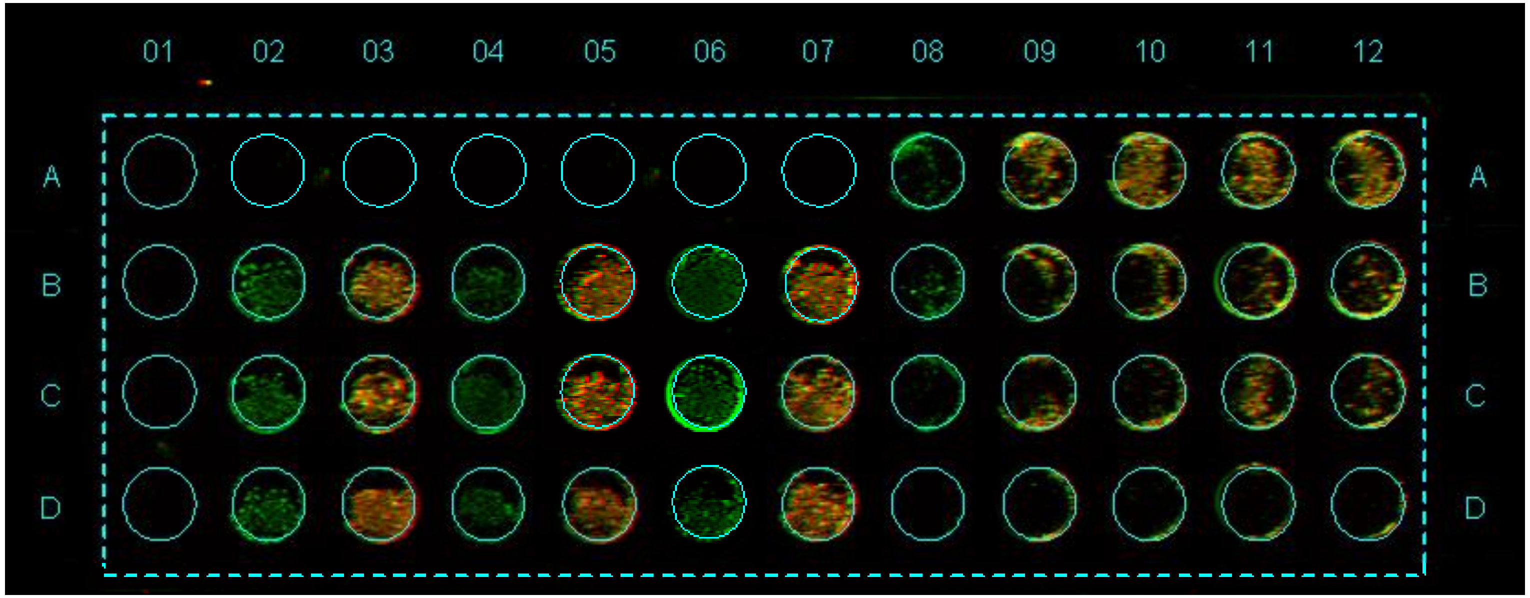

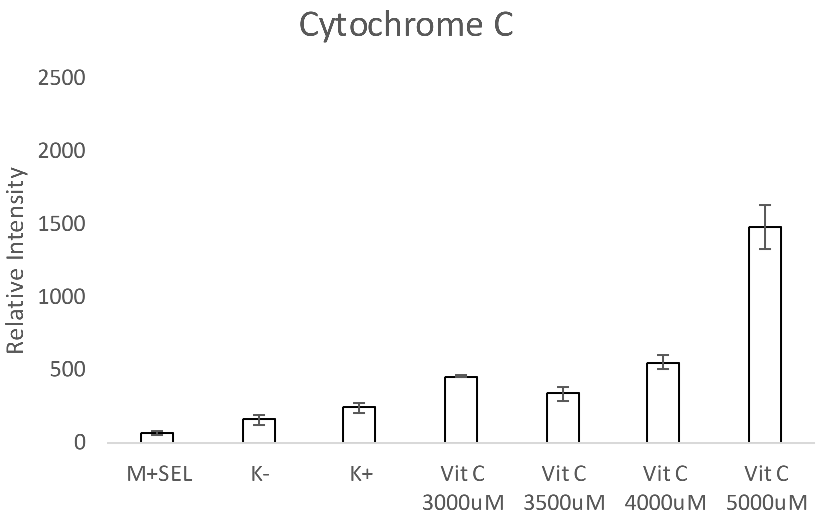

BLANK	BLANK	BLANK	BLANK	BLANK	BLANK	BLANK	BCK Vit C 3000uM	Vit C 3000uM	Vit C 3000uM	Vit C 3000uM	Vit C 3000uM
BLANK	BCK K-	K-	BCK K+	K+	BCK M+SEL	M+SEL	BCK Vit C 3500uM	Vit C 3500uM	Vit C 3500uM	Vit C 3500uM	Vit C 3500uM
BLANK	BCK K-	K-	BCK K+	K+	BCK M+SEL	M+SEL	BCK Vit C 4000uM	Vit C 4000uM	Vit C 4000uM	Vit C 4000uM	Vit C 4000uM
BLANK	BCK K-	K-	BCK K+	K+	BCK M+SEL	M+SEL	BCK Vit C 5000uM	Vit C 5000uM	Vit C 5000uM	Vit C 5000uM	Vit C 5000uM

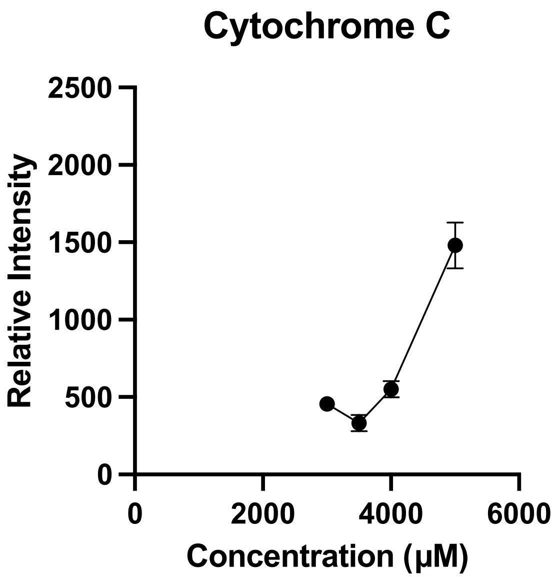

	**Cytochrome C**	**SEM**
**M+SEL**	67,01412378	14,59410315
**K-**	156,1175849	32,76246425
**K+**	236,6143049	38,80606833
**Vit C 3000uM**	454,8170728	9,97963012
**Vit C 3500uM**	332,5001777	53,31678342
**Vit C 4000uM**	550,9729713	52,02659863
**Vit C 5000uM**	1479,173071	148,7092753

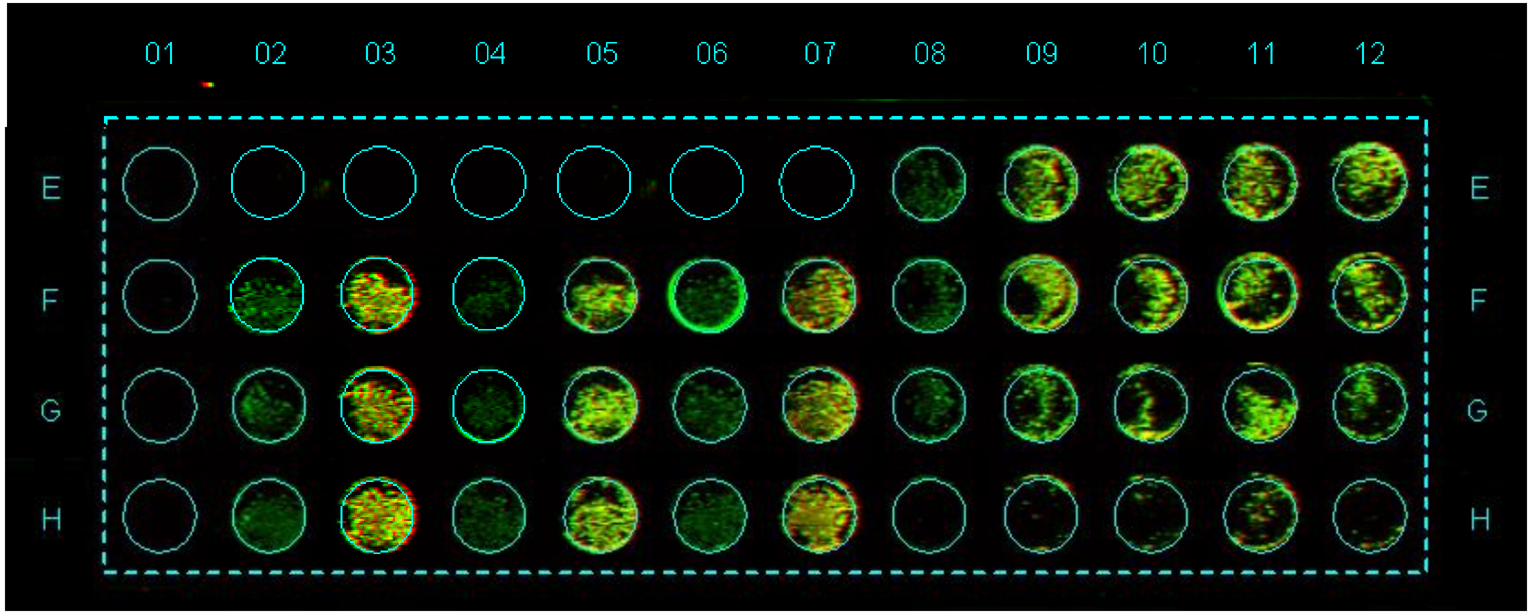

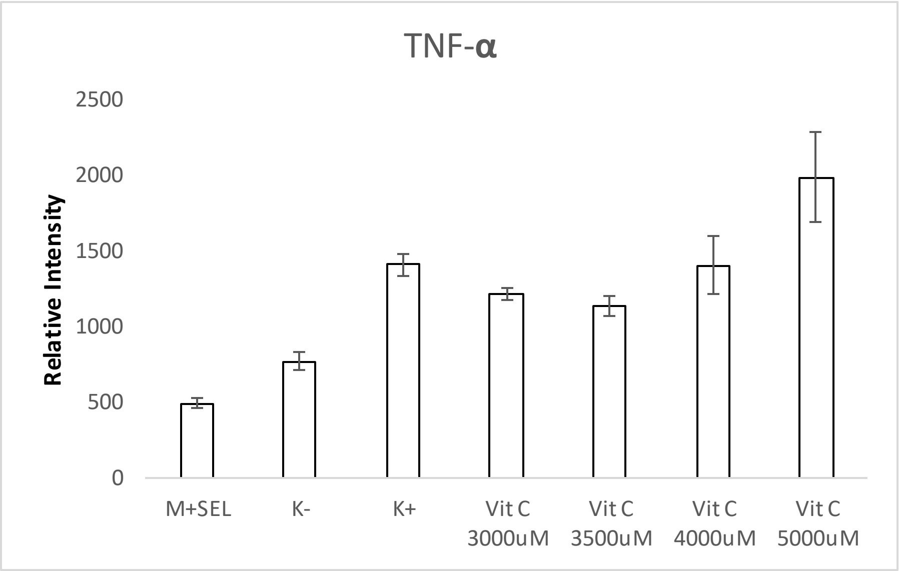

BLANK	BLANK	BLANK	BLANK	BLANK	BLANK	BLANK	BCK Vit C 3000uM	Vit C 3000uM	Vit C 3000uM	Vit C 3000uM	Vit C 3000uM
BLANK	BCK K-	K-	BCK K+	K+	BCK M+SEL	M+SEL	BCK Vit C 3500uM	Vit C 3500uM	Vit C 3500uM	Vit C 3500uM	Vit C 3500uM
BLANK	BCK K-	K-	BCK K+	K+	BCK M+SEL	M+SEL	BCK Vit C 4000uM	Vit C 4000uM	Vit C 4000uM	Vit C 4000uM	Vit C 4000uM
BLANK	BCK K-	K-	BCK K+	K+	BCK M+SEL	M+SEL	BCK Vit C 5000uM	Vit C 5000uM	Vit C 5000uM	Vit C 5000uM	Vit C 5000uM

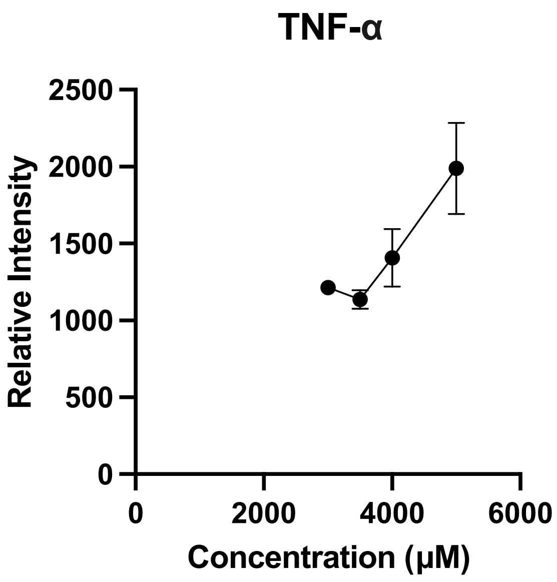

	TNF-α	SEM
**M+SEL**	492,0481096	34,94132918
**K-**	769,5734079	58,81185203
**K+**	1410,839105	76,39157718
**Vit C 3000uM**	1214,157131	36,9447365
**Vit C 3500uM**	1137,118821	61,71002517
**Vit C 4000uM**	1407,31956	188,0637838
**Vit C 5000uM**	1988,991745	297,2905753

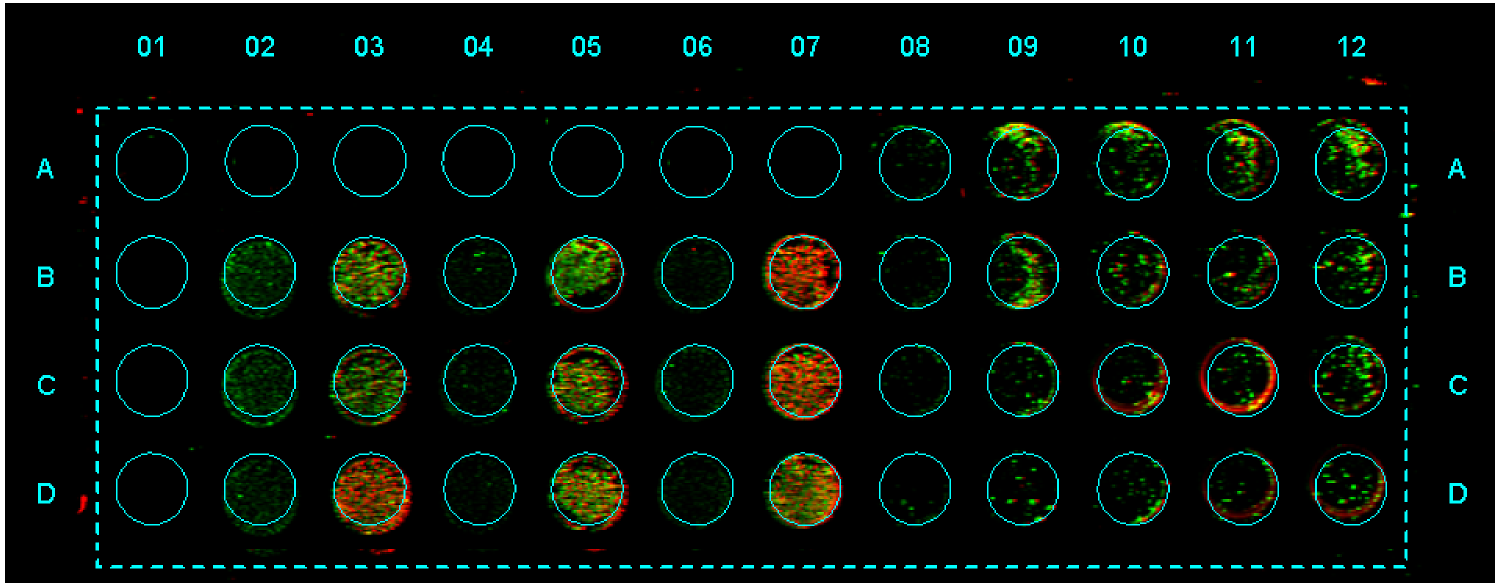

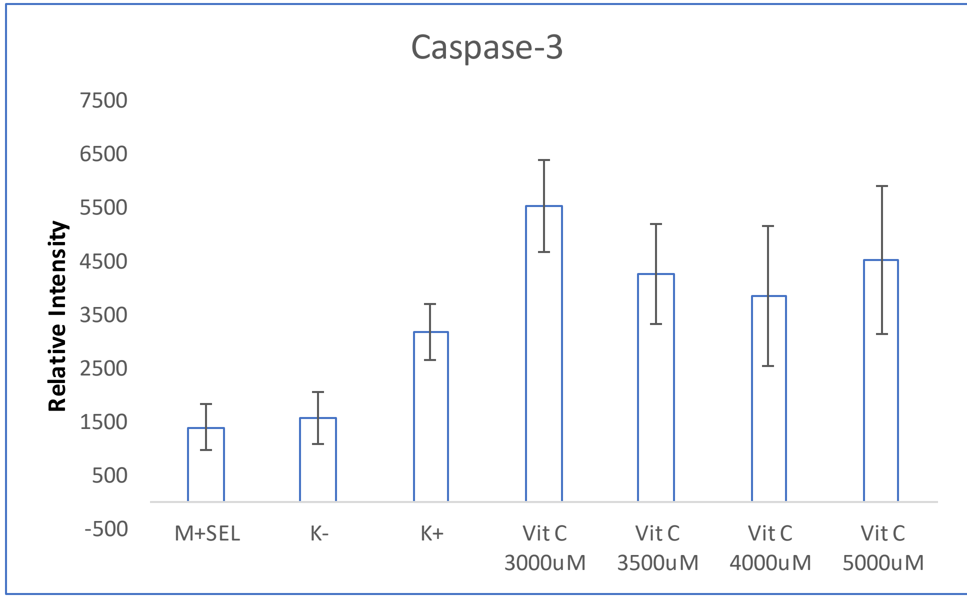

BLANK	BLANK	BLANK	BLANK	BLANK	BLANK	BLANK	BCK Vit C 3000uM	Vit C 3000uM	Vit C 3000uM	Vit C 3000uM	Vit C 3000uM
BLANK	BCK K-	K-	BCK K+	K+	BCK M+SEL	M+SEL	BCK Vit C 3500uM	Vit C 3500uM	Vit C 3500uM	Vit C 3500uM	Vit C 3500uM
BLANK	BCK K-	K-	BCK K+	K+	BCK M+SEL	M+SEL	BCK Vit C 4000uM	Vit C 4000uM	Vit C 4000uM	Vit C 4000uM	Vit C 4000uM
BLANK	BCK K-	K-	BCK K+	K+	BCK M+SEL	M+SEL	BCK Vit C 5000uM	Vit C 5000uM	Vit C 5000uM	Vit C 5000uM	Vit C 5000uM

	Caspase 3	SEM
M+SEL	1407,371938	429,1329001
K-	1581,139399	495,3710742
K+	3188,673848	532,042819
Vit C 3000uM	5544,812527	862,4264849
Vit C 3500uM	4262,971364	932,9925122
Vit C 4000uM	3872,72751	1309,777256
Vit C 5000uM	4532,494394	1378,154193

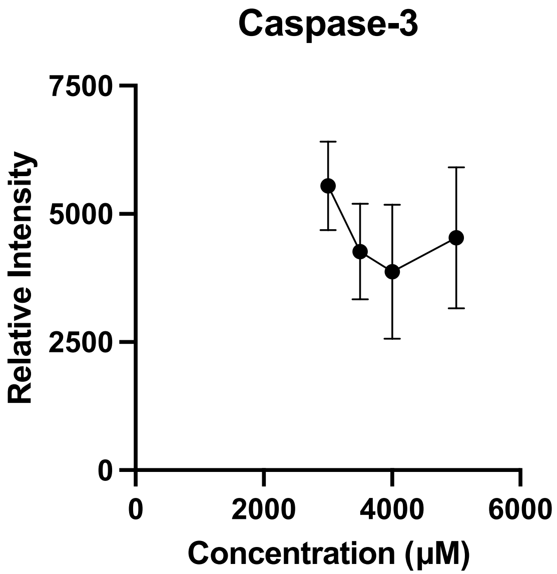

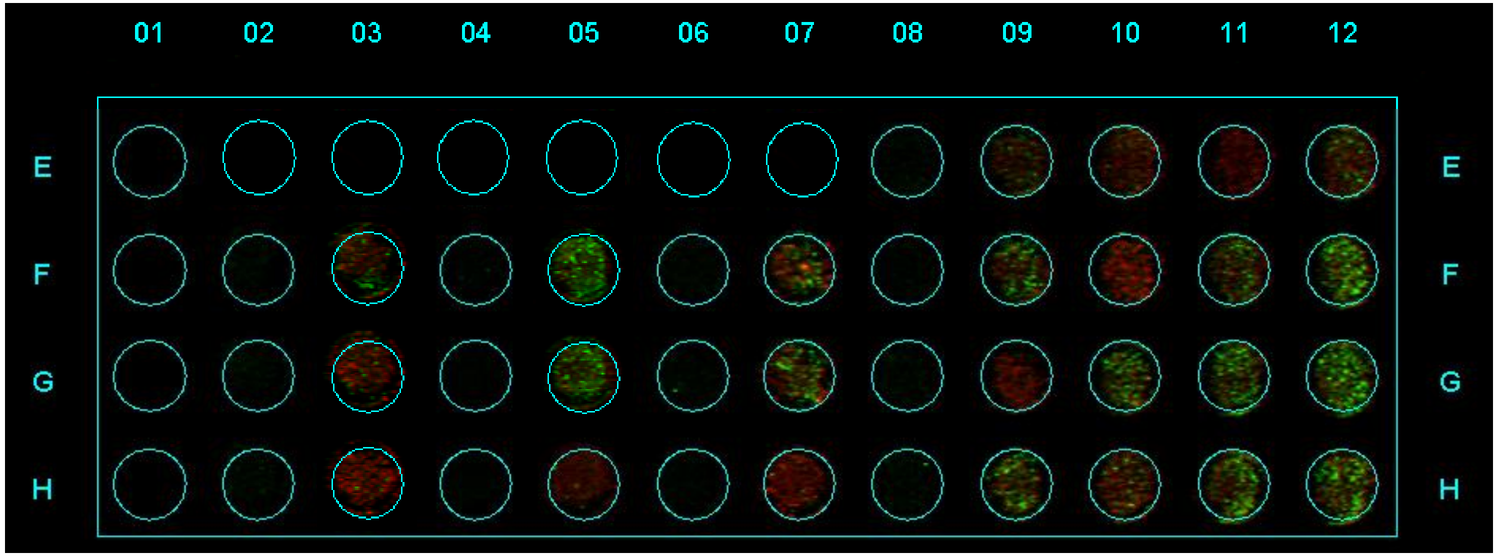

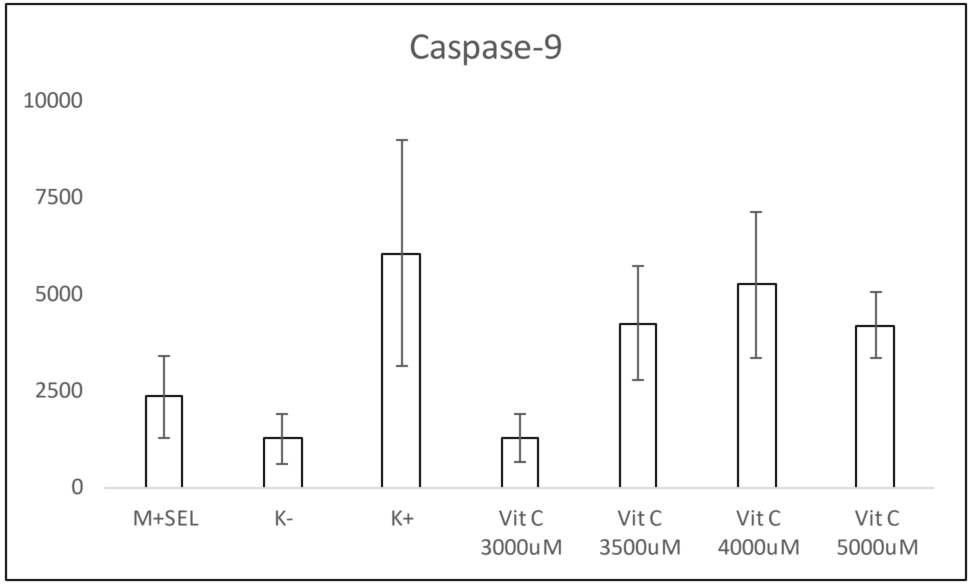

BLANK	BLANK	BLANK	BLANK	BLANK	BLANK	BLANK	BCK Vit C 3000uM	Vit C 3000uM	Vit C 3000uM	Vit C 3000uM	Vit C 3000uM
BLANK	BCK K-	K-	BCK K+	K+	BCK M+SEL	M+SEL	BCK Vit C 3500uM	Vit C 3500uM	Vit C 3500uM	Vit C 3500uM	Vit C 3500uM
BLANK	BCK K-	K-	BCK K+	K+	BCK M+SEL	M+SEL	BCK Vit C 4000uM	Vit C 4000uM	Vit C 4000uM	Vit C 4000uM	Vit C 4000uM
BLANK	BCK K-	K-	BCK K+	K+	BCK M+SEL	M+SEL	BCK Vit C 5000uM	Vit C 5000uM	Vit C 5000uM	Vit C 5000uM	Vit C 5000uM

	Caspase-9	SEM
**M+SEL**	2342,810736	1052,323601
**K-**	1259,039792	642,0106946
**K+**	6064,265644	2911,653739
**Vit C 3000uM**	1291,359338	611,3510923
**Vit C 3500uM**	4255,942807	1484,537181
**Vit C 4000uM**	5247,396707	1886,682708
**Vit C 5000uM**	4193,919126	847,8465556

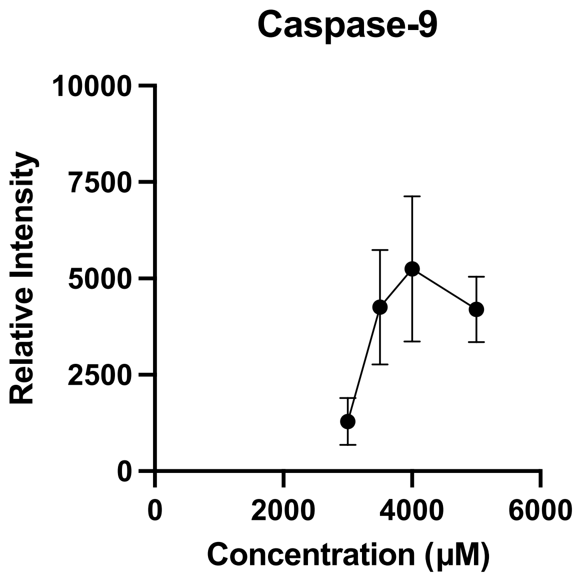

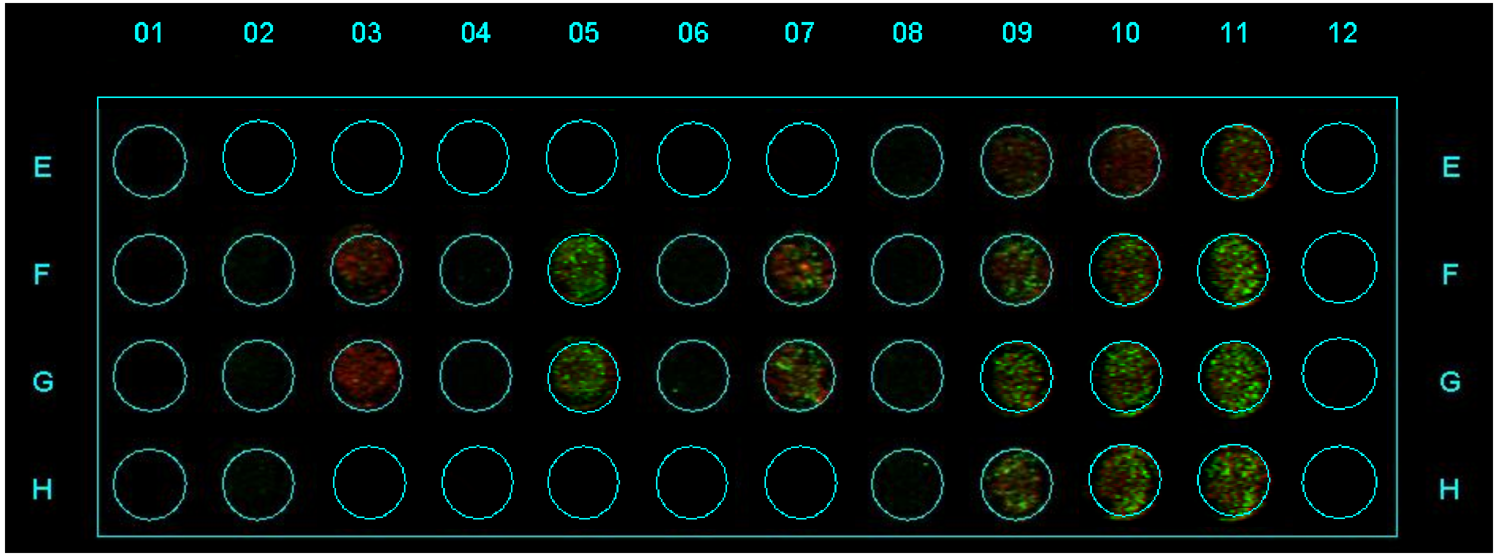

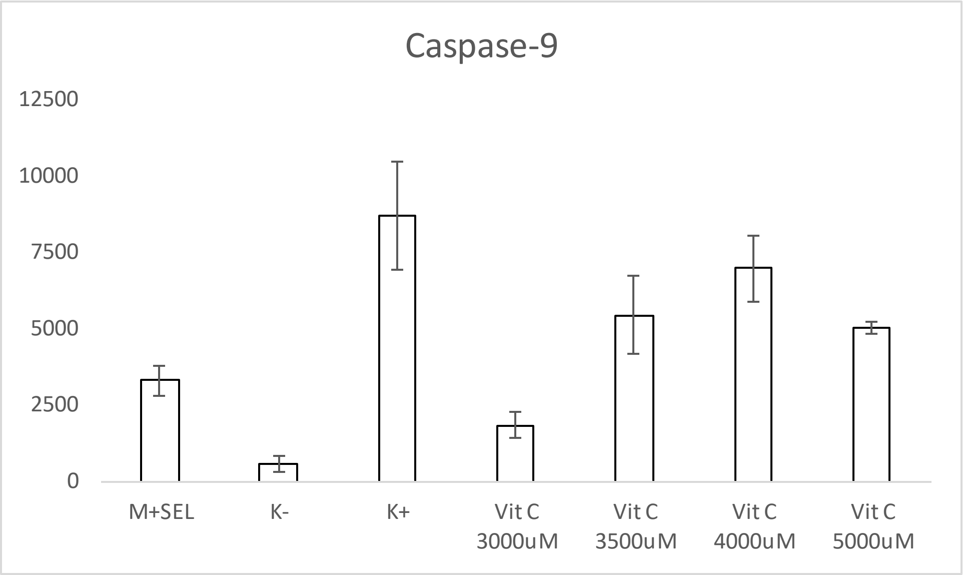

BLANK	BLANK	BLANK	BLANK	BLANK	BLANK	BLANK	BCK Vit C 3000uM	Vit C 3000uM	Vit C 3000uM	Vit C 3000uM
BLANK	BCK K-	K-	BCK K+	K+	BCK M+SEL	M+SEL	BCK Vit C 3500uM	Vit C 3500uM	Vit C 3500uM	Vit C 3500uM
BLANK	BCK K-	K-	BCK K+	K+	BCK M+SEL	M+SEL	BCK Vit C 4000uM	Vit C 4000uM	Vit C 4000uM	Vit C 4000uM
BLANK	BLANK	BLANK	BLANK	BLANK	BLANK	BLANK	BCK Vit C 5000uM	Vit C 5000uM	Vit C 5000uM	Vit C 5000uM

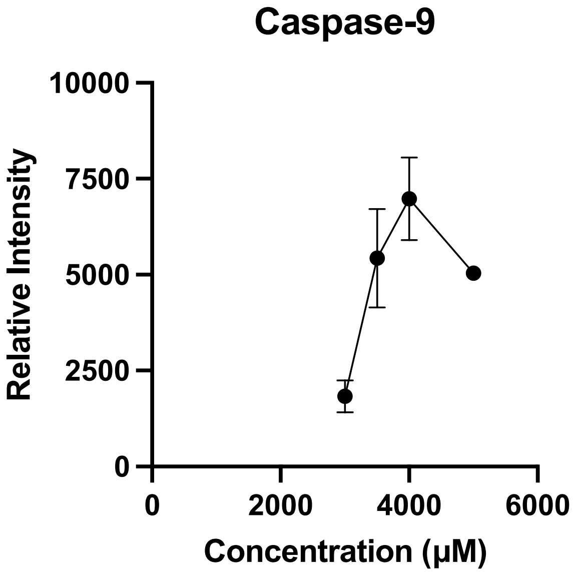

	Caspase-9	SEM
**M+SEL**	3293,938578	491,0589393
**K-**	543,4452843	252,3170469
**K+**	8721,555355	1765,122062
**Vit C 3000uM**	1827,8918	417,9563971
**Vit C 3500uM**	5433,106766	1286,865544
**Vit C 4000uM**	6978,412051	1077,467121
**Vit C 5000uM**	5033,626113	197,6752796

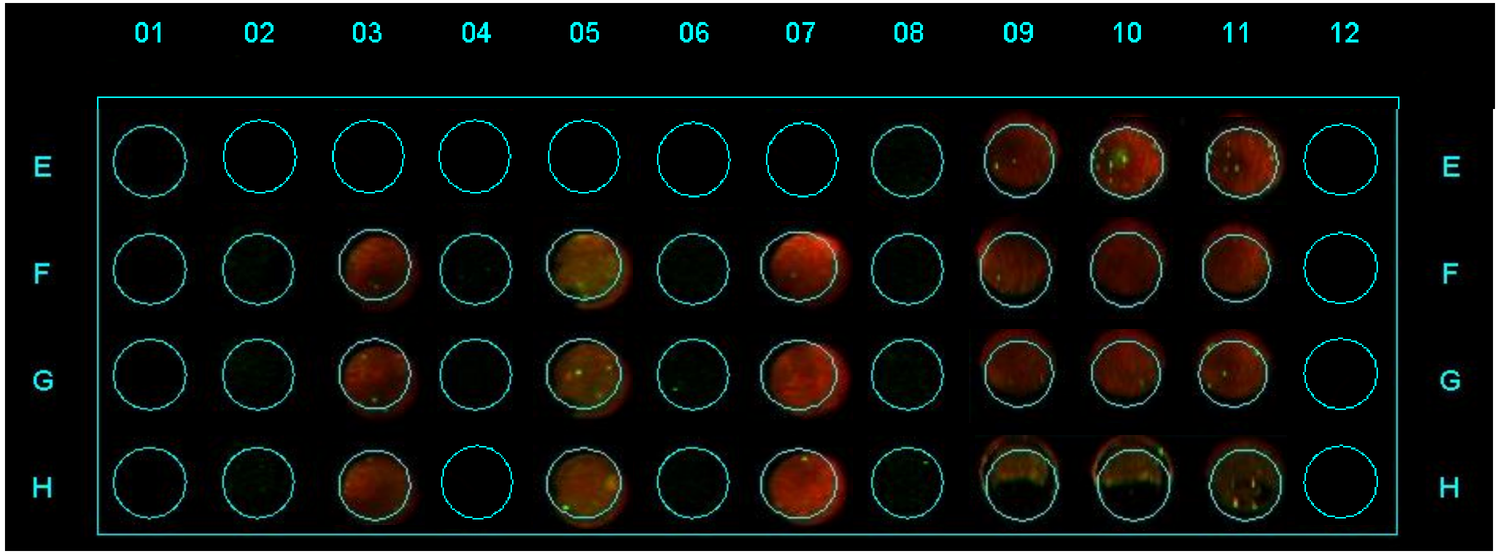

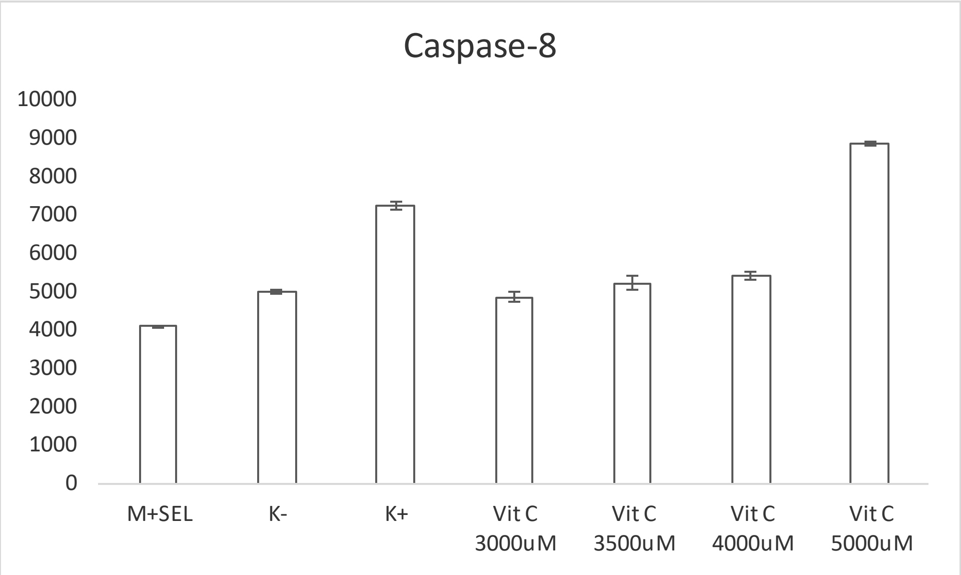

BLANK	BLANK	BLANK	BLANK	BLANK	BLANK	BLANK	BCK Vit C 3000uM	Vit C 3000uM	Vit C 3000uM	Vit C 3000uM	Vit C 3000uM
BLANK	BCK K-	K-	BCK K+	K+	BCK M+SEL	M+SEL	BCK Vit C 3500uM	Vit C 3500uM	Vit C 3500uM	Vit C 3500uM	Vit C 3500uM
BLANK	BCK K-	K-	BCK K+	K+	BCK M+SEL	M+SEL	BCK Vit C 4000uM	Vit C 4000uM	Vit C 4000uM	Vit C 4000uM	Vit C 4000uM
BLANK	BCK K-	K-	BCK K+	K+	BCK M+SEL	M+SEL	BCK Vit C 5000uM	Vit C 5000uM	Vit C 5000uM	Vit C 5000uM	Vit C 5000uM

	Caspase-8	SEM
M+SEL	4094,889959	17,70852431
K-	5011,062787	58,19572835
K+	7258,179223	111,0379199
Vit C 3000uM	4852,998388	141,2416159
Vit C 3500uM	5228,66981	201,3057751
Vit C 4000uM	5417,379282	103,6727535
Vit C 5000uM	8858,416951	58,83630217

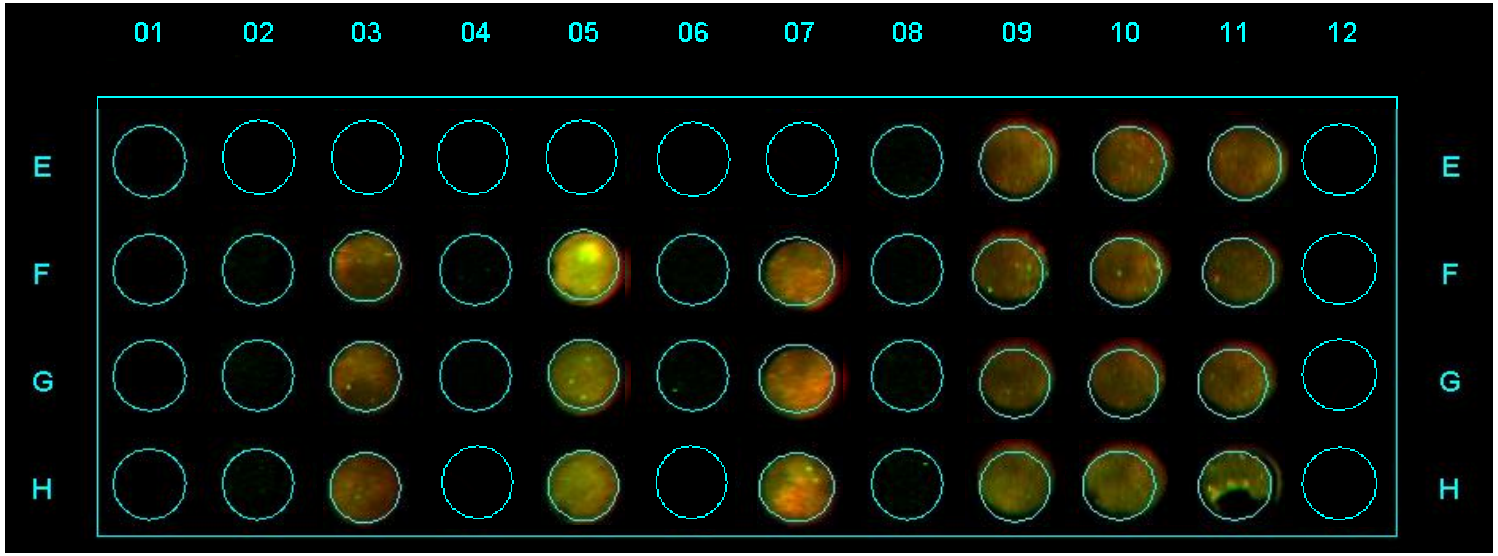

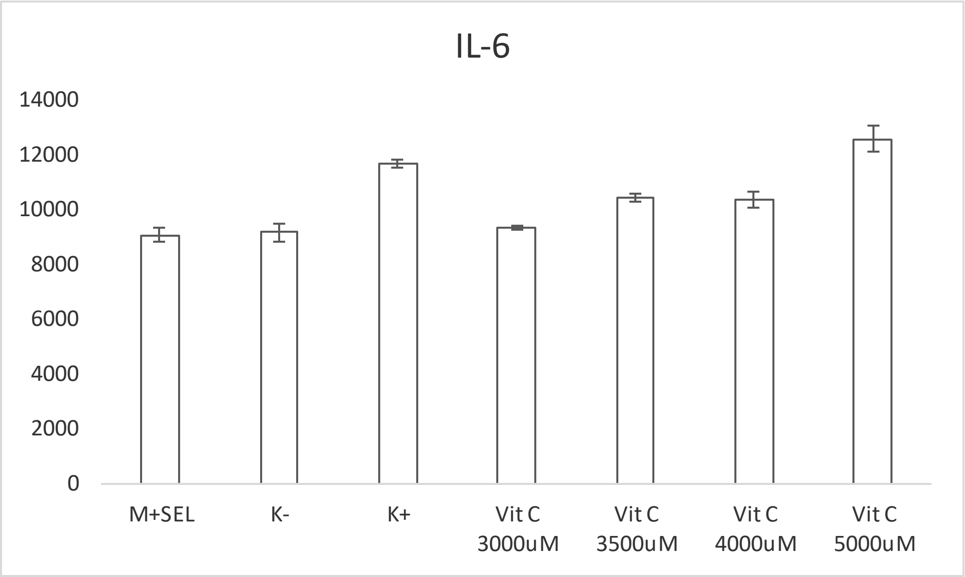

BLANK	BLANK	BLANK	BLANK	BLANK	BLANK	BLANK	BCK Vit C 3000uM	Vit C 3000uM	Vit C 3000uM	Vit C 3000uM	Vit C 3000uM
BLANK	BCK K-	K-	BCK K+	K+	BCK M+SEL	M+SEL	BCK Vit C 3500uM	Vit C 3500uM	Vit C 3500uM	Vit C 3500uM	Vit C 3500uM
BLANK	BCK K-	K-	BCK K+	K+	BCK M+SEL	M+SEL	BCK Vit C 4000uM	Vit C 4000uM	Vit C 4000uM	Vit C 4000uM	Vit C 4000uM
BLANK	BCK K-	K-	BCK K+	K+	BCK M+SEL	M+SEL	BCK Vit C 5000uM	Vit C 5000uM	Vit C 5000uM	Vit C 5000uM	Vit C 5000uM

	IL-6	SEM
M+SEL	9088,97726	267,296555
K-	9190,12267	336,6853548
K+	11688,69879	156,5273921
Vit C 3000uM	9376,950064	78,80084758
Vit C 3500uM	10445,73499	147,2235009
Vit C 4000uM	10378,56414	265,0336217
Vit C 5000uM	12600,8866	453,9131664

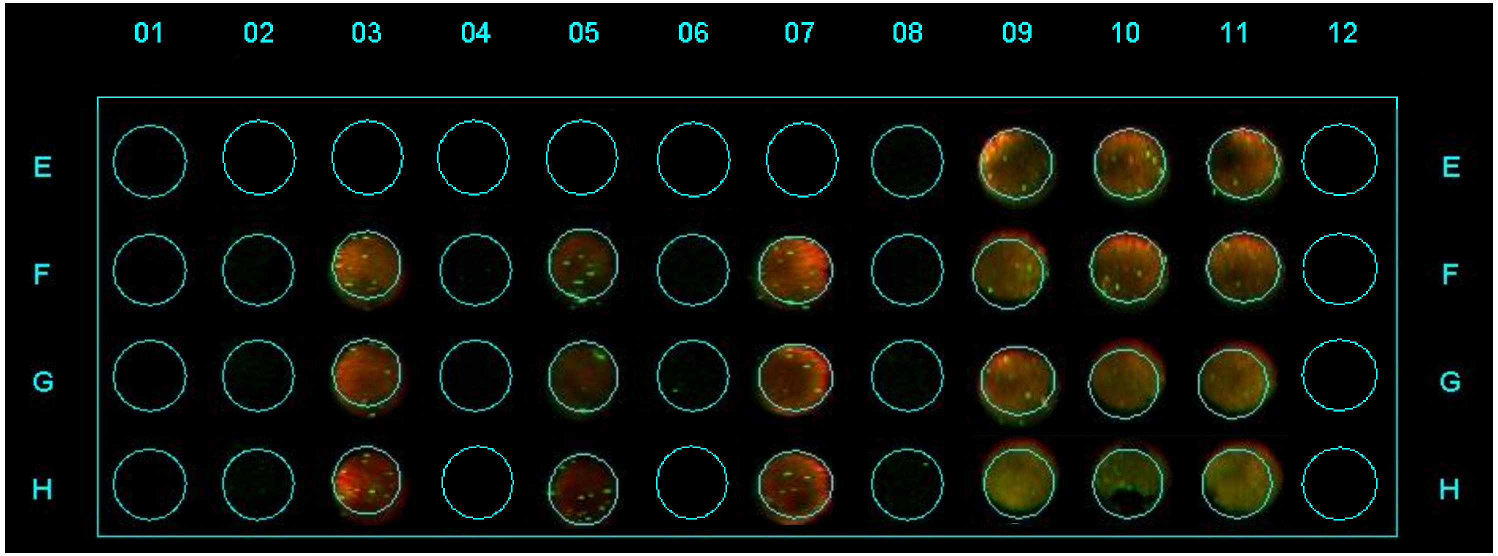

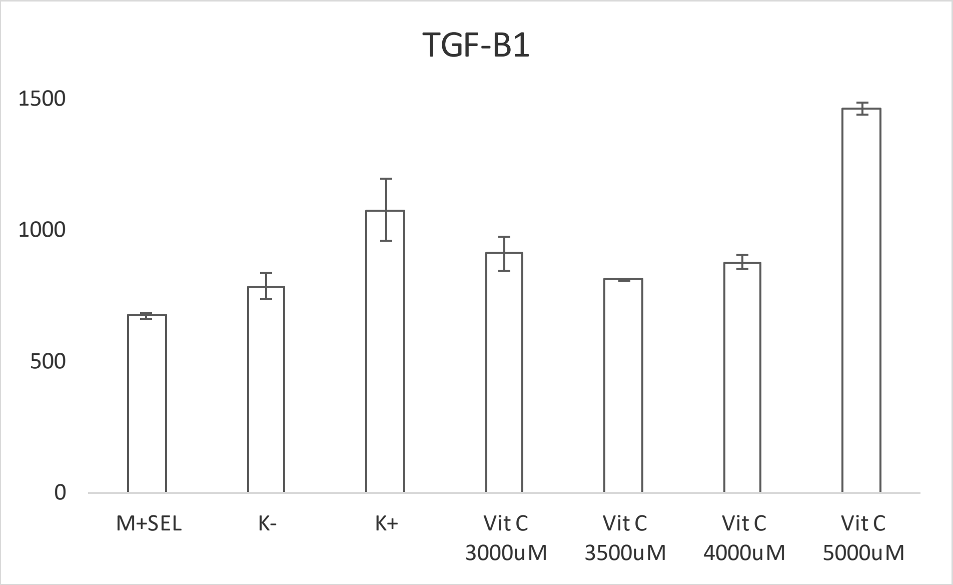

BLANK	BLANK	BLANK	BLANK	BLANK	BLANK	BLANK	BCK Vit C 3000uM	Vit C 3000uM	Vit C 3000uM	Vit C 3000uM	Vit C 3000uM
BLANK	BCK K-	K-	BCK K+	K+	BCK M+SEL	M+SEL	BCK Vit C 3500uM	Vit C 3500uM	Vit C 3500uM	Vit C 3500uM	Vit C 3500uM
BLANK	BCK K-	K-	BCK K+	K+	BCK M+SEL	M+SEL	BCK Vit C 4000uM	Vit C 4000uM	Vit C 4000uM	Vit C 4000uM	Vit C 4000uM
BLANK	BCK K-	K-	BCK K+	K+	BCK M+SEL	M+SEL	BCK Vit C 5000uM	Vit C 5000uM	Vit C 5000uM	Vit C 5000uM	Vit C 5000uM

	TGF-B1	SEM
M+SEL	675,6649476	9,058995603
K-	787,6810403	48,34846431
K+	1078,697839	117,8053386
Vit C 3000uM	912,1943915	66,5840969
Vit C 3500uM	815,1315246	4,101861613
Vit C 4000uM	879,9673645	26,16787128
Vit C 5000uM	1463,087	22,42283417

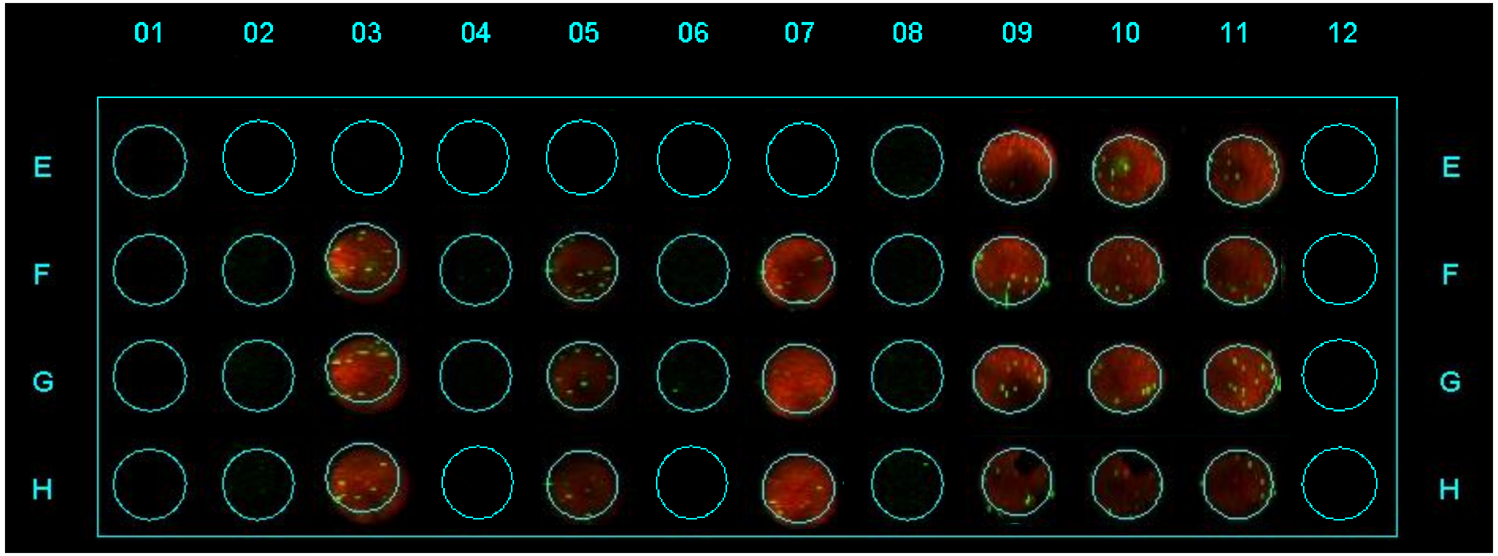

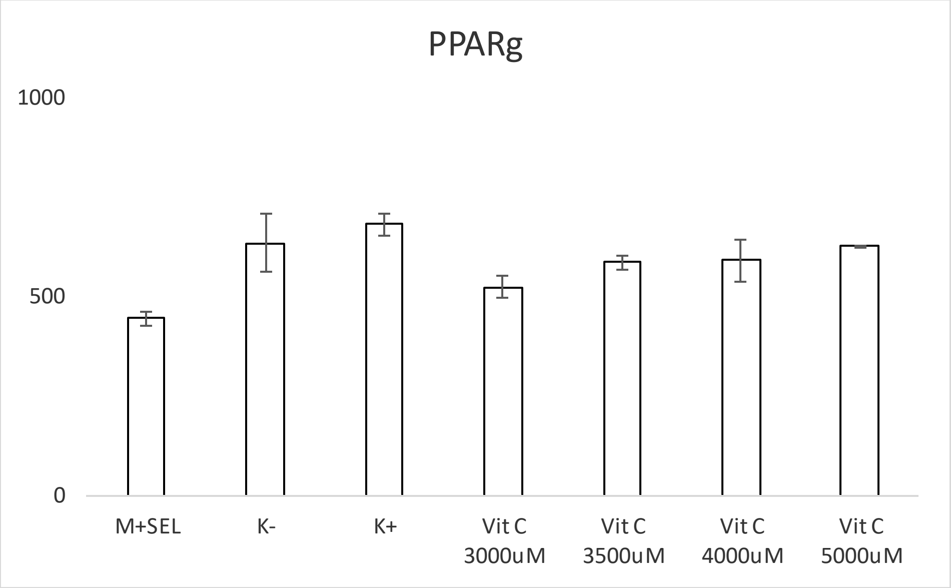

BLANK	BLANK	BLANK	BLANK	BLANK	BLANK	BLANK	BCK Vit C 3000uM	Vit C 3000uM	Vit C 3000uM	Vit C 3000uM	Vit C 3000uM
BLANK	BCK K-	K-	BCK K+	K+	BCK M+SEL	M+SEL	BCK Vit C 3500uM	Vit C 3500uM	Vit C 3500uM	Vit C 3500uM	Vit C 3500uM
BLANK	BCK K-	K-	BCK K+	K+	BCK M+SEL	M+SEL	BCK Vit C 4000uM	Vit C 4000uM	Vit C 4000uM	Vit C 4000uM	Vit C 4000uM
BLANK	BCK K-	K-	BCK K+	K+	BCK M+SEL	M+SEL	BCK Vit C 5000uM	Vit C 5000uM	Vit C 5000uM	Vit C 5000uM	Vit C 5000uM

	PARPg	SEM
M+SEL	445,760781	16,60186428
K-	634,3932001	72,94639658
K+	683,3492758	26,87494084
Vit C 3000uM	524,4781428	28,06793783
Vit C 3500uM	586,7221623	16,70058627
Vit C 4000uM	592,0208866	53,88193139
Vit C 5000uM	629,2121225	1,577608616
